# Skin-Resident *γδ* T Cells Exhibit Site-Specific Morphology and Activation States

**DOI:** 10.1155/2019/9020234

**Published:** 2019-01-06

**Authors:** Abigail S. Marshall, Jaqueline Raymondi Silva, Courtney A. Bannerman, Ian Gilron, Nader Ghasemlou

**Affiliations:** ^1^Queen's University, Departments of Biomedical & Molecular Sciences, Kingston, ON, Canada; ^2^Queen's University, Departments of Anesthesiology & Perioperative Medicine, Kingston, ON, Canada; ^3^Queen's University, Centre for Neuroscience Studies, Kingston, ON, Canada

## Abstract

Skin-resident *γδ* T cells play an important role in maintaining the immune barrier at the epithelial surface. Their roles in wound healing, regulation of immune response to injury, and reepithelialization have been characterized extensively in the mouse, though their function in human skin remains largely unknown. Human skin-resident *γδ* T cells sparsely populate the skin and are often small and rounded in appearance. Those in the mouse ear and back, which line the dermal barrier, are highly arborized cells with many processes extending from the cell body. To date, these cells have been studied primarily in the mouse ear and back; however, it is important to further identify and characterize *γδ* T cells in other body sites to better understand their function and study their contribution to injury and disease. We developed a novel method to visualize these cells in the skin (whole-mount and cryosections) that when combined with flow cytometry allowed us to assess differences in skin-resident *γδ* T cell numbers, morphology, and activation state in the ear, back, and footpad (chosen for their importance in immunological and pain research). In comparing cell length, number of dendritic processes, and expression of the activation marker CD69, we found that *γδ* T cell morphology and activation states vary significantly among the three tissue environments. Specifically, *γδ* T cells in the footpad are smaller, have fewer processes, and show the highest levels of activation compared to back- and ear-resident cells. Our observations suggest that our understanding of skin-resident *γδ* T cell functionality, drawn from the experiments performed in the ear and back tissue, may not be applicable to all skin environments. The footpad-resident cells also more closely resemble *γδ* T cells in human skin, suggesting that cells in this tissue environment may serve as a better translational model when studying *γδ* T cell function/activity.

## 1. Introduction

*γδ* T cells are critical to the maintenance and regulation of the immune barrier at epithelial surfaces in mammals, despite their relatively limited role in systemic T cell immunity. Since their identification over 30 years ago thanks to the cloning of the *γ* T cell receptor [1–5], elucidating the specific biology and physiological roles of *γδ* T cells in murine skin has remained an active area of investigation. *γδ* T cells make up only a small percentage of circulating CD3+ lymphocytes in mice, with lymphocytes bearing the *αβ* T cell receptor (TCR) being far more prevalent. This paradigm is reversed in murine skin, where *γδ* T cells make up the majority (>50%) of skin-resident CD3+ T lymphocytes [6, 7]. Skin-resident *γδ* T cells interact extensively with keratinocytes via their dendritic processes [8] and are critical for maintenance of skin homeostasis via insulin-like growth factor 1 (IGF-1), wound healing via keratinocyte growth factors, and the initiation of the proliferative phase of healing after burn injury [9–12]. Thus, *γδ* T cell-derived factors appear to play a significant role in maintaining the protective physical and immune barrier of the epithelial microenvironment in murine skin. However, most of these experiments were carried out in the back or ear tissue where these cells are plentiful. In human skin, *γδ* T cells are infrequent and do not have the same dendritic appearance as they do in the mouse [13–15].

Immunofluorescent staining of skin-resident *γδ* T cells using a PE-conjugated antibody in epithelial sheets demonstrate that these cells have a distinctive dendritic morphology at rest [9, 10]. After injury (or other noxious insult to the skin), *γδ* T cells proximal to the wound edge adopt a rounded morphology and transiently lose their dendrites [9, 16]. Adoption of the rounded morphology is accompanied by heightened expression of the cell surface glycoprotein CD69, which is a well-characterized marker of *γδ* T cell activation [17]. Current immunohistochemical methods used to identify skin-resident *γδ* T cells are either cost-prohibitive or do not provide adequate in situ visualization of localization and morphology. We therefore sought to develop a novel immunofluorescence technique for the effective visualization of these cells through the full thickness of the skin (dermis and epidermis inclusive), without necessitating investment in TCR*δ*-transgenic mice expressing eGFP or Cre-recombinase [18, 19].

Through developing a new strategy for immunostaining *γδ* T cells, we examined the morphology and activation of these cells in specific skin niches including two often-studied sites in immunology (the ear and back) and one important for pain research (the footpad). Behavioural outcomes such as mechanical and thermal sensitivity are measured primarily through stimulation of the footpad [20, 21], making this an important site for studying neuroimmune interaction, nociception, and pain; the proximity of these *γδ* T cells to sensory fibers in the skin would also suggest a potential role in the pain response. Our work suggests a significant difference in absolute numbers, morphology, and activation state of *γδ* T cells across the three skin sites and that where *γδ* T cells reside may be critical for their function and contribution to both homeostatic and inflammatory conditions.

## 2. Methods

### 2.1. Experimental Animals

Male 6–12-week-old C57BL/6J mice (Jackson Labs, Bar Harbor, ME) were used in all experiments. Mice were housed in a temperature- and humidity-controlled room on a 12-hour light-dark cycle with free access to food and water. All animals were cared for under experimental protocols approved by Queen's University Animal Care Committee and in accordance with the Canadian Council on Animal Care guidelines.

### 2.2. Whole-Mount Immunofluorescence

Mice were deeply anaesthetized using sodium pentobarbital (Bimeda MTC, Cambridge, ON, Canada) and transcardially perfused with 25–30 ml ice cold paraformaldehyde (PFA) in 0.1 M phosphate buffer (PB). PFA was used at 0, 2, or 4%. Hindlimbs, ears, and back skin were removed using microscissors. Back skin was shaved and treated with Veet depilatory cream (Reckitt Benckiser, Massy, France) for 5 minutes in an effort to reduce autofluorescence caused by hair. All samples were postfixed in 0, 2, or 4% PFA for one hour at 4°C. Skin from footpads was excised from the ankle joint to the base of the toes and was separated from the muscle and subcutaneous fat. The two layers of the ear were separated mechanically using forceps, and the intervening fatty tissue gently removed. All samples were washed in 1X phosphate-buffered saline (PBS) for 20 minutes at 4°C.

Tissues were then immersed in a solution of StartingBlock Pre-Made Blocking Buffer (Thermo Fisher Scientific, Waltham, MA) with 2% TritonX-100 and 0.1% sodium azide in 1.5 ml Eppendorf tubes overnight at room temperature. Samples were then incubated in Image-iT FX signal enhancer (Thermo Fisher Scientific) on glass slides for three hours at room temperature, followed by overnight incubation at room temperature in Armenian hamster anti-mouse TCR*δ* primary antibody (1 : 100; Invitrogen, Carlsbad, CA). The tissue was then washed in a 0.1% sodium azide in PBS solution three times for one hour each time at room temperature and then incubated overnight in FITC-conjugated goat anti-Armenian hamster IgG at room temperature (1 : 200; BioLegend, San Diego, CA). Both primary and secondary antibodies were diluted in the StartingBlock/TritonX/sodium azide solution.

Samples were again washed twice for one hour and once overnight in 0.1% sodium azide in PBS solution (7–10 ml). Tissue was then mounted on glass slides and incubated with Vectashield Antifade Mounting Medium containing DAPI (Vector Labs, Burlingame, CA) for two hours. Slides were then covered with glass coverslips, sealed using nail polish, and stored at 4°C until ready for visualization.

### 2.3. Frozen Cryosection Immunofluorescence

Tissue was collected as previously described (see “Whole-mount immunofluorescence” above) and cryoprotected in 30% sucrose solution at 4°C. Tissue was rapidly frozen in Tissue Freezing Medium (Leica Biosystems, Wetzlar, Germany) and stored at −80°C. Fifteen-micron thick sections were obtained using a Leica CM1860 cryostat at −20°C. Slides were stored at −20°C until staining. When ready for immunostaining, slides were warmed to room temperature and washed twice in PBS with 0.25% TritonX-100 for ten minutes. Sections were then incubated with 300 *μ*l of a 10% normal serum (5% donkey and 5% goat) blocking solution in PBS with 0.2% TritonX-100 for one hour, then washed twice for 10 minutes each time in PBS with 0.2% TritonX-100. Slides were then incubated overnight in Armenian hamster anti-mouse TCR*δ* primary antibody (1 : 100, as in whole-mount immunohistochemistry), then washed in 1X PBS three times, for ten minutes each time at room temperature. Tissue was then incubated with the secondary antibody (1 : 200, as in whole-mount immunohistochemistry) for two hours at room temperature. Slides were washed again with 1X PBS three times, for ten minutes each time, and mounted using Vectashield DAPI-containing mounting medium and covered with a glass coverslip. Tissue was visualized using a Zeiss AxioSkop2 (Carl Zeiss AG, Oberkochen, Germany) fluorescent microscope, with images acquired using the Zeiss AxioVision software.

### 2.4. Morphological Analysis

Quantification of *γδ* T cell morphology was carried out by assessing cell length and number of processes per cell. Cell length was measured using MetaMorph software (Molecular Devices, San Jose, CA) calibrated using micrometer images from the fluorescent microscope used for image acquisition. Cellular processes were counted manually as numbers of branches emanating from the cell body. Sections were analyzed in *n* = 3-5 animals, with ≥116 cells analyzed per tissue site.

### 2.5. Flow Cytometry

Mice were deeply anaesthetized with sodium pentobarbital and sacrificed by cervical dislocation. Skin was harvested from the ear, footpad, and back, with hair removed from the back using a razor and Veet depilatory cream, as described above. Cell suspensions were prepared from skin sections and stained with the following antibodies: PE-conjugated anti-CD3, APC/Fire-750-conjugated anti-TCR*γδ*, and FITC-conjugated anti-CD69 (1 : 200, all antibodies from BioLegend, San Diego, CA). Samples were run on a CytoFlex flow cytometer (Beckman Coulter, Brea, CA) and analysis performed using CytExpert software (Beckman Coulter).

### 2.6. Statistical Analysis

Results were analyzed using SigmaPlot (Systat Software, Inc., San Jose, CA), with one-way ANOVA analysis and *post-hoc* Tukey test (threshold set at *p* < 0.05) performed for all group comparisons. Data are reported as mean ± SEM.

## 3. Results

### 3.1. Immunohistochemical Analysis of *γδ* T Cells across Skin Sites

To better study the *γδ* T cells in the skin, we focused on identifying a methodology by which we can visualize the cells across various tissue sites. Sections of skin from the ear, back, and footpad were taken from animals perfused with either 0.1 M phosphate buffer (0%), 2% paraformaldehyde, or 4% paraformaldehyde and immunostained using the GL3 clone of anti-TCR*γ*/*δ* for whole-mount immunohistochemistry. We found that *γδ* T cells were not detected in mice perfused with PBS or 4% paraformaldehyde (data not shown), while cells were clearly observed in skin with 2% paraformaldehyde.

**Figure 1:**
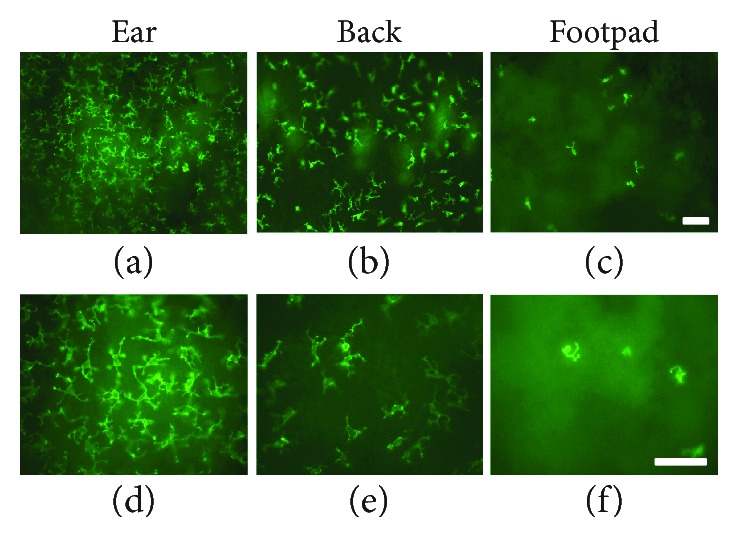
Fluorescent immunohistochemical analysis of skin-resident *γδ* T cells in whole-mount tissue. Skin obtained from the murine ear, back, and footpad was stained using anti-TCR*γ*/*δ* antibody and visualized using whole-mount immunofluorescence. There appear to be greater numbers of *γδ* T cells, with more stellate morphology, in the ear (a, d) and back (b, e) than in the footpad (c, f). Cells in the footpad have a more rounded appearance, with fewer processes emanating from the cell body. Scale bars = 50 *μ*m.

**Figure 2:**
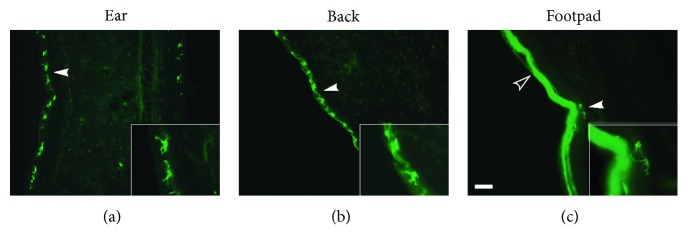
Fluorescent immunohistochemical analysis of skin-resident *γδ* T cells in cryosectioned tissue. Frozen cryosections of skin obtained from the murine ear, back, and footpad were stained using anti-TCR*γ*/*δ* antibody and visualized using cryosection immunofluorescence. As in whole-mount sections, *γδ* T cells in the skin show variable morphological characteristics dependent on skin site. TCR*γ*/*δ*-immunopositive cells (indicated by white arrows) are found primarily in the epidermis of the ear (a), back (b), and footpad (c). There is an increased background staining in the epidermis of the footpad (indicated by black arrow), though cells are not found in this area. Insets are enlarged representations of *γδ* T cells from the three skin sites. Scale bar = 50 *μ*m.

Whole-mount immunostaining of *γδ* T cells in the ear and back showed similar morphology to that described before [7, 9, 10, 16], with numerous processes extending from the cell body and ending in densely stained boutons (Figure 1). In the footpad, however, *γδ* T cells showed a distinctly different morphology, with a more rounded appearance and fewer processes stemming from the cell body. Tissues collected from mice perfused as above were also used for frozen cryosectioning. As with whole-mount sections, *γδ* T cells were only visualized following perfusion with 2% paraformaldehyde using the same antibody, and not when perfused with PBS or 4% paraformaldehyde. Cryosectioned skin showed evidence of *γδ* T cells primarily in the epidermal layer across all skin sites (Figure 2). There was equivocal staining in the dermal layer of the ear and back, which may be suggestive of the presence of dermal *γδ* T cells (see white arrow, Figure 2(a)); however, elevated background staining in the footpad impedes the effective visualization and definitive identification of *γδ* T cells in the dermis (Figure 2). Similar to whole-mount tissue, processes are seen arising from *γδ* T cells in the ear and back and less so from those in the footpad. Specificity of this new staining method was confirmed using TCRδ^+/+^ and TCR*δ* ^−/−^ ears immunostained with the TCR*δ* antibody (Supplemental Figure 1).

### 3.2. Morphological Analysis of *γ*δ T Cells across Skin Sites

**Figure 3:**
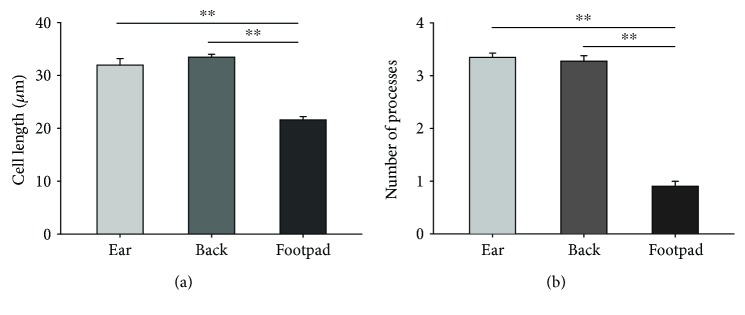
Morphological assessment reveals a significant variability in numbers of processes and size of *γδ* T cells across three tissue sites. (a) Cell length, measured as the greatest distance between two branches, was measured in *γδ* T cells in the ear, back, and footpad. As evidenced by their rounder morphology, cells in the footpad were significantly smaller in length than those in the ear or back. (b) The number of processes emanating from the cell body was counted in *γδ* T cells in the ear, back, and footpad. Cells in the ear and back have a significantly greater number of processes than *γδ* T cells in the footpad. Bar graphs represent mean ± SEM (^∗∗^ *p* < 0.001, one-way ANOVA with *post-hoc* Tukey test; *n* = 3-5/group with ≥116 cells analyzed per site).

Our qualitative observations of *γδ* T cells led us to quantify the morphology of the cells in the three skin sites examined. We first calculated total cell length (*n* = 120-348 cells per site), measured as the longest linear distance between two branches on a cell, and found significant differences between the three sites (Figure 3(a); one-way ANOVA, *p* < 0.001, *n* = 3-5 mice per group). The mean cell length in the ear and back was significantly larger than in the footpad owing to the rounded shape of the cells, with ear and back cells 31.9 ± 1.2 and 33.4 ± 0.6 *μ*m in length, respectively, while footpad cells were 21.6 ± 0.6 *μ*m (Figure 3(b); *p* < 0.001, one-way ANOVA with *post-hoc* Tukey test). No differences were observed in the length between ear and back *γδ* T cells (*p* = 0.482, one-way ANOVA with *post-hoc* Tukey test).

We next quantified the number of branches/projections arising from *γδ* T cells (*n* = 116-218 cells per site) and observed significant differences between the three sites examined (Figure 3(b); one-way ANOVA, *p* < 0.001, *n* = 3-5 per group). *γδ* T cells in the footpad had significantly fewer processes than those in the ear or back, as suggested by their round shape (*p* < 0.001, one-way ANOVA with *post-hoc* Tukey test; footpad: 0.93 ± 0.08; ear: 3.35 ± 0.08, and back: 3.28 ± 0.10 processes per cell). No differences were observed in the number of processes between the back and ear (*p* = 0.866, one-way ANOVA with *post-hoc* Tukey test).

### 3.3. Differential *γδ* T Cell Numbers and Activation States across Skin Sites

**Figure 4:**
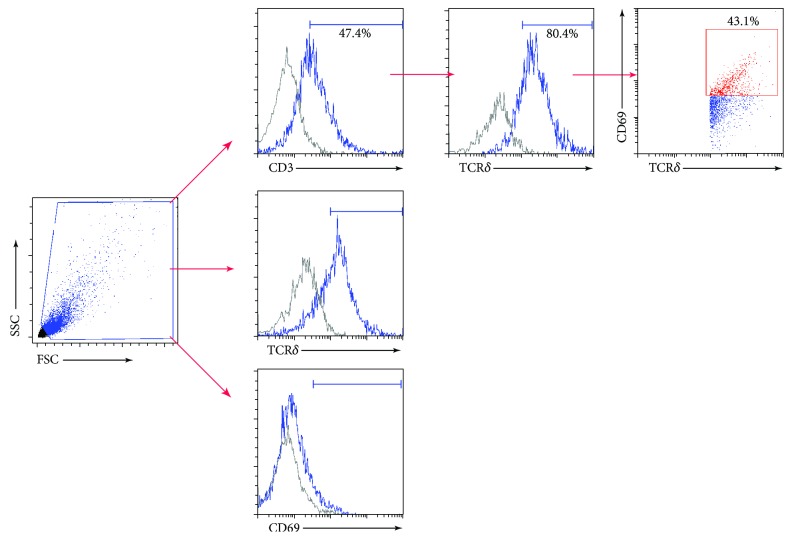
Gating strategy for *γδ* T cells. Single-cell suspensions were first gated for physical parameters, including forward scatter (FSC), a measure of size, and side scatter (SSC), a measure of cell granularity. *γδ* T cells were selected as positive for expression of CD3, a coreceptor expressed by all T cells, followed by gating on those also expressing TCR*γ*/*δ*, represented as shaded areas. Activated *γδ* T cells were identified based on their increased expression of CD69. Appropriate isotype controls are shown in gray with stained samples in blue.

**Figure 5:**
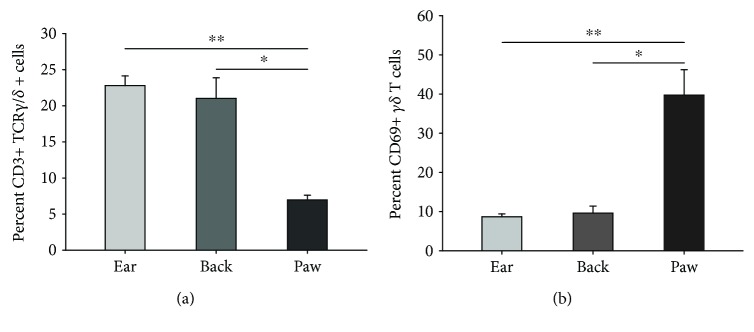
Flow cytometry reveals a significant variation in *γδ* T cell numbers and activation states across three skin sites. There are significantly fewer *γδ* T cells, which also show increased CD69 expression, in the footpad relative to the ear and back. (a) The percentage of CD3+ cells expressing TCRγ/δ is significantly greater in the ear and back than in the footpad. (b) The activation state of CD3 + TCR*γ*/*δ* + cells, assessed using the cell-surface antigen CD69, shows increased expression among cells in the footpad than in the ear and back. Bar graphs represent mean ± SEM (^∗∗^ *p* < 0.001 and ^∗^ *p* < 0.01, one-way ANOVA with *post-hoc* Tukey test; *n* = 4/group).

We noticed striking differences in the total number of *γδ* T cells across the three sites, with few cells evident in the footpad. Published data (using tissue taken from the ear) suggests that *γδ* T cells make up more than half of CD3+ lymphocytes in murine skin [7]. We therefore used flow cytometry to quantify the proportion of *γδ* T cells across the three sites, as a percentage of all CD3+ cells, to determine whether this observation was consistent in the footpad as well. Cells were sorted based on their coexpression of CD3 and TCRγ/δ receptors and classified as the *γδ* T cell population in skin from the ear, back, and footpad (see Figure 4). The mean percentage of CD3 + TCR*γ*/*δ* + cells varied significantly between ear, back, and footpad skin (*n* = 4 per group, one-way ANOVA, *p* < 0.001; Figure 5(a)). Approximately 20% of all CD3+ cells in the ear and back skin were *γδ* T cells, while only 7% were identified in the footpad (one-way ANOVA with *post-hoc* Tukey test, *p* ≤ 0.001).

Cells in the naïve uninjured footpad are more rounded and with fewer processes than those in the ear or back, suggestive of an activated profile [9]; we therefore used CD69 to assess activation states of the cells and found significant differences across the three sites (Figure 5(b); *p* < 0.001, one-way ANOVA). While fewer than 10% of all *γδ* T cells were CD69+ in the ear and back, 40% were immune-positive for the marker in the footpad (*p* ≤ 0.001, one-way ANOVA with *post-hoc* Tukey test).

## 4. Discussion

We developed an immunofluorescent staining method which allows for the effective visualization of skin-resident *γδ* T cells both in whole-mount and in serial cryosections without the need for custom antibodies or transgenic mice. These methods have facilitated the morphological characterization of these cells in three different skin environments in the mouse: the ear, back, and footpad. The ear and back were chosen as these are sites often used for the study of *γδ* T cells, while the footpad was chosen since it is most often used in pain research to assess changes in hypersensitivity. Our results show that *γδ* T cells vary significantly in their morphology depending on their skin niche. Specifically, *γδ* T cells in the ear and back were longer and had more dendritic processes extending from the cell body than did footpad-resident cells. As evidenced in both our histological sections and by flow cytometry, *γδ* T cells in the footpad were more sparsely distributed throughout the tissue than in the ear and back. Using flow cytometry, we also demonstrate that a greater percentage of CD3 + TCR*γ*/*δ*+ cells in the footpad express the activation marker CD69 than do cells in the ear or back, indicating that *γδ* T cells in this specific niche exist at a higher basal level of activation than in other skin regions.

Immunohistochemical and flow cytometric analysis of skin-resident T cells in humans has shown that they are primarily composed of *αβ* T cells, with *γδ* T cells making up a considerably smaller proportion of all CD3+ cells than is observed in rodents [15, 22, 23]. While it remains unknown why such differences exist between the two species, functional studies assessing the role of *γδ* T cells in the mouse have been carried out almost exclusively in the ear or back where these cells exist in relatively high numbers. Given the distinct variation in *γδ* T cell morphology and activation states across skin environments that we have observed, it may be important to assess the contribution of *γδ* T cells to skin injury and disease across different skin sites. Further, morphological parameters of footpad-resident *γδ* T cells suggest that these cells are morphologically similar to human *γδ* T cells [13–15]. The *αβ*/*γδ* T cell ratio in the footpad also better models the lymphocyte ratio in human skin. Thus, our results suggest that *γδ* T cells in the rodent footpad may serve as a relevant site to study *γδ* T cells to infer function in human skin and allow for more complete phenotypic characterization of *γδ* T cell physiology. While we suggest using the footpad as an improved translational model to study skin-resident *γδ* T cells, use of the ear or back will be critical to study the mechanisms through which these cells interact with other immune cells and their environment. The increased autofluorescence present in the footpad, due to the increased keratin present, makes the visualization of *γδ* T cells more difficult. This is a drawback to the technique developed. Use of the ear and back has provided an important insight into the varied physiological roles of these cells. Indeed, the abundance of *γδ* T cells in ear and back skin likely amplifies effects that may not have been determinable or significant in paw-resident *γδ* T cells but are nonetheless important functions of these cells. We therefore propose that future studies include paw skin in addition to skin from other environments to allow for more complete phenotypic characterization of their function and physiological properties.

One important question that arises from our work is why skin-resident *γδ* T cells are phenotypically distinct in the footpad relative to the ear and back. We propose two potential avenues: either the microenvironment is distinct among the three sites or the increased activity in the footpad results in the sustained activation and loss of *γδ* T cells. In support of the differences in tissue environment, there is evidence that immune cell numbers, pigmentation, and innervation are distinct across the three sites. For instance, analysis of human skin samples across three body sites found distinct differences in epidermal thickness, pigmentation, and blood content [24]. Other groups have observed similar patterns, as well as differences in the morphology of keratinocytes and dermal papillae [25, 26]. Sensory and motor innervation of the skin have also been found to directly alter epidermal thickness [27] and are also different across various skin sites in humans [28, 29]. Immune cell numbers in the human skin, such as that of mast cells [30, 31] and lymphocytes [32], also show variations across skin sites. Interestingly, Foster and colleagues [32] found that *αβ* T cell numbers were greatest in the sole of the foot than in all other sites examined; *γδ* T cell numbers were not found to be different across any sites examined in this same study. The footpad has a much thicker epidermal layer with increased keratin at the apical surface (as evidenced by the increased autofluorescence in our tissue samples). Whether this contributes to the differences observed remains unknown.

The differences observed in the footpad may also be due to their sustained use and activity relative to the ears and back skin of the mice. The wood chip bedding used in our animal care facility provides a hard surface that may cause constant irritation or activation of the footpad. Sensory neurons, which are known to secrete growth factors, cytokines, and neuropeptides in response to injury/disease [33–35], may cause the early activation of *γδ* T cells. This overactivation of the cells may ultimately lead to their loss or downregulation of cell-surface markers (e.g., CD3, TCR*γ*/*δ*). Understanding these differences may shed new light on skin-resident *γδ* T cell maturation and survival.

## 5. Conclusions

We utilized a novel immunofluorescence method (in concert with flow cytometry) to visualize *γδ* T cells in the skin. This allowed us to identify, for the first time, distinct morphological and activation phenotypes depending on the tissue environment in which these cells reside. Our choice to include the footpad was based on our goal of assessing the role of skin-resident *γδ* T cells in nociception and inflammatory pain, but now provides a potentially new avenue to translate the findings of these important cells to human injury and disease. Our immunofluorescence method may also be useful for assessing time-course changes in *γδ* T cell activation after injury. Further, as the immunological role of skin-resident γδ T cells continues to be explored, this method may also prove useful in visualizing these cells in gut, genital, and oral mucosa as well.

## Data Availability

The immunohistochemistry and flow cytometry data used to support the findings of this study are included within the article. Any additional data required are available from the corresponding author.

## Additional Points

*Highlights*. (i) A method is described for whole-mount/cryosection immunostaining of *γδ* T cells. (ii) Presence, morphology, and activation states of *γδ* T cells is site-specific in mouse skin (ear, back, and footpad). (iii) Cells are smaller, with fewer branches, and higher CD69 expression in the footpad. (iv) *γδ* T cells in mouse footpad more closely resemble human skin-resident cells.

## Supplementary Materials

Supplementary Materials Figure S1: specificity of immunostaining for TCRδ was confirmed using sections of ears from wild-type TCRδ+/+ and TCRδ−/− (knockout) mice. While *γδ* T cells are observed in the epidermal layer in wild-type mice (a; arrow), the cells are absent in sections from knockout mice (b).
