# Supplementary material for: Skin-Resident γδ T Cells Exhibit Site-Specific Morphology and Activation States
Source: J Immunol Res. 2019 Jan 6;2019:9020234. doi: 10.1155/2019/9020234 (PMC6339708; doi:10.1155/2019/9020234)
Supplement: Supplementary Materials — Figure S1: specificity of immunostaining for TCRδ was confirmed using sections of ears from wild-type TCRδ+/+ and TCRδ−/− (knockout) mice. While γδ T cells are observed in the epidermal layer in wild-type mice (a; arrow), the cells are absent in sections from knockout mice (b). [file 9020234.f1.pdf]

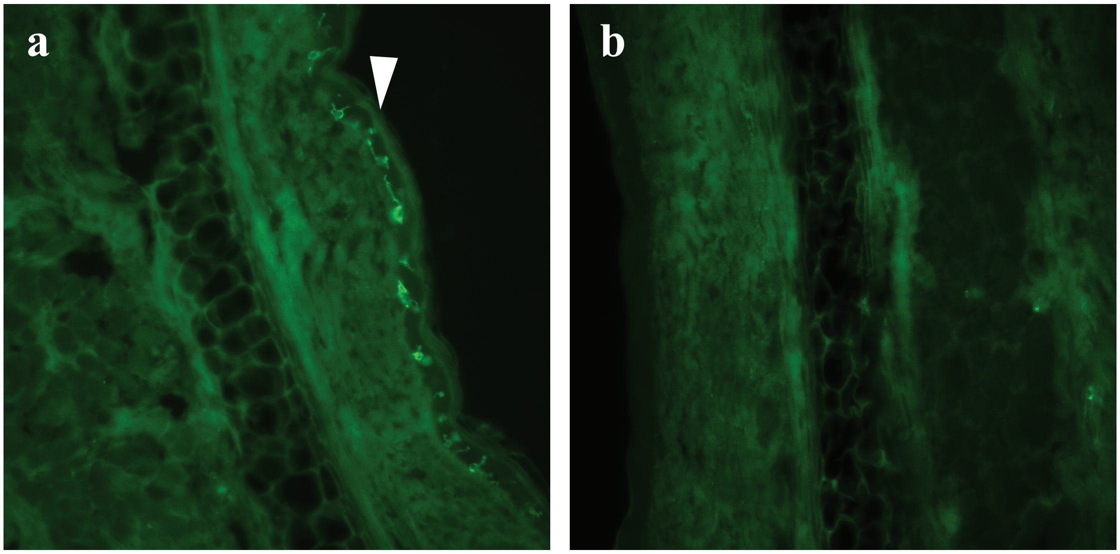

Supplemental Figure 1. Specificity of immunostaining for TCRδ was confirmed using sections of ears from wild-type TCRδ+/+ and TCRδ-/- (knockout) mice. While γδ T cells are observed in the epidermal layer in wild-type mice (a; arrow), the cells are absent in sections from knockout mice (b).
